# The effects of monetary policies on foreign direct investment inflows in emerging economies: some policy implications for post-COVID-19

**DOI:** 10.1186/s43093-022-00152-6

**Published:** 2022-09-23

**Authors:** Özcan Karahan, Musa Bayır

**Affiliations:** Bandırma Onyedi Eylül Univesity, Bandırma, Balıkesir Turkey

**Keywords:** Foreign direct investment, Stock market index, Interest rate, Pandemics

## Abstract

Expansionary monetary policies, which started to be implemented after the global crisis in 2008 and became widespread during the COVID-19 period, lowered global interest rates and increased the stock market indexes. This study aims to investigate the effects of expansionary monetary policies implemented before and during COVID-19 on foreign direct investment (FDI) flows to emerging economies. In this context, the effect of expansionary monetary policies on FDI has been tried to be determined through the changes created in financial indicators such as interest rate and stock market index. Accordingly, the effects of the developments in the global stock market index and interest rates on the FDI for Brazil, China, Turkey, and Poland were estimated using the autoregressive distributed lag (ARDL) model for the period 2008–2021. Empirical findings show that expansionary monetary policy practices before and during COVID-19 causing high global stock market index and low-interest rates encourage FDI inflows to developing countries. The empirical results on the effects of expansionary monetary policies applied before and during COVID-19 on FDI allow important implications to be made when considered together with the contractionary monetary policies to be implemented after COVID-19. So much so that our empirical results in favour of FDI flows to developing countries regarding the expansionary monetary policies implemented before and during COVID-19 imply that the transition to contractionary monetary policy in the post-COVID-19 period may cause significant constraints on the FDI inflows to developing countries. Therefore, it may be expected that favourable financial conditions for foreign direct capital inflows to developing countries will disappear in the post-COVID-19 period. In other words, the falling global stock market index and increasing interest rates along with the contractionary monetary policies implemented in the post-COVID-19 period will be able to have the potential to cause a significant change in the investment preferences of international companies towards developing economies. The general policy prescription obtained from the results of the study shows that developing countries would need much more FDI-attracting policies in order to compensate for the negative financial effects of contractionary monetary policies implemented in the post-COVID-19 period.

## Introduction

Foreign Direct Investment (FDI) is considered an important component of their development strategy for emerging economies. Accordingly, policymakers give huge significance to the design of policy to stimulate inward FDI flows. FDI not only provides developing countries the much-needed capital but also enhances employment and production as well as the transfer of technology. Besides, some other benefits for the host country have been identified like the introduction of improved management techniques, international trade integration, and the evolution of a more competitive business environment. FDI has also been more stable or less risky compared to other capital flows like portfolio investments and debt flows [43]: 9–19). Although FDI creates an important opportunity for the development of developing countries in many ways, it is seriously affected by the fluctuations in the global economy. Sometimes shocks in the world economy prevent the effective spread of FDI around the world and reduce the benefits for developing countries.

With the global economic crisis experienced in 2008, unconventional monetary policy practices are widely applied all over the world. In this way, expansionary monetary policy tools have started to be used more powerfully and widely than ever before. The fact that Central Banks have the opportunity to respond to the crisis more quickly by increasing liquidity compared to fiscal policy instruments has led to the implementation of expansionary monetary policy by a wide group of countries. Accordingly, after the 2008 crisis, the Federal Reserve System (FED) adopted a series of unconventional policies as an alternative to traditional measures to stimulate aggregate demand and restore the functioning of the financial system. In addition, with the COVID-19 epidemic that started in China in 2019 and spread all over the world, the economies experienced another great shock. Thereupon, almost all countries continued to implement expansionary monetary policies more vigorously in order to eliminate the negative effects of the COVID-19. Thus, the expansionary monetary policy, which started to be implemented in 2008 with the global crisis, was also widely adopted throughout the world during the COVID-19 period.

Due to the expansionary monetary policies implemented before and during COVID-19, significant changes were created in global financial indicators such as the interest rate and stock market index. The abundance of liquidity, which became abundant with the expansionary monetary policies, decreased the interest rates and increased the stock market indexes at a global level. It has been revealed by many studies in the literature that the expansionary monetary policies implemented by the central banks in this period decreased the interest rates and increased the stock market index [7]; [11, 37], [8].

The aim of this study is to reveal the impact of expansionary monetary policies applied before and during the COVID-19 on FDI inflows to developing countries. In this framework, it will be tried to determine how the applied expansionary monetary policies affect the amount of FDI towards emerging markets through the changes in the interest rate and stock market index. Accordingly, data from emerging markets such as Brazil, China, Turkey and Poland between the years 2008–2021 were analysed using the ARDL method. The examined countries were chosen to represent examples of developing countries in different continents of the world.

In the framework of empirical analysis, we try to determine not only the basic financial dynamic of FDI flows to emerging markets before and during COVID-19 but also policies needed in order to sustain the inflow of FDI to developing countries in the post-COVID 19 period. In the last period, with the loss of importance of the COVID-19 and inflation increases, it seems that most of the central banks have started to return to contractionary monetary policies. Of course, the drastic change in monetary policies implemented before and during the COVID-19 in the upcoming post-COVID-19 period will undoubtedly have a profound impact on the FDI inflows to developing countries. Therefore, the findings of this study will enable us to make some inferences about the possible effects of the contractionary monetary policies implemented in the post-COVID-19 period on FDI flows to developing countries.

The rest of the paper proceeds as following plan. Section “Literature review” reviews the main related literature. Section “Data and methodology” presents data, methodology, and empirical results. The final section concludes and makes some policy implications in order to maintain stable FDI inflows to the emerging market.

## Literature review

There has been a significant increase in foreign direct investment because of the world becoming a single global village with globalization. Especially, FDI has increasingly become an important source of economic growth and development for emerging economies. In addition to increasing employment and production in developing countries, FDI has also led to significant changes in the production structure through information and technology diffusion. For this reason, developing countries all over the world have made a great effort to attract foreign direct investments to their countries. Due to the increasing importance of FDI in the world economy in terms of developing economies, the factors affecting the FDI trend have begun to be studied extensively in the economics literature.

In order for multinational companies to invest abroad, certain conditions that will increase their profit expectations must be met. In the literature, besides the global economic conjuncture, many factors related to the economic situation of both the home country and the foreign country in which the multinational company will invest have been determined. While an important part of these factors is related to real economic indicators, another important part considers financial indicators.

Indeed, looking at the literature it seems that determinants of FDI movements are closely tied to emerging countries’ real and financial-economic conditions. Accordingly, in reviewing the empirical evidence it is crucial to distinguish between the real and financial factors driving capital flows to emerging markets. This distinction is important because the drivers of capital flows differ crucially depending on the real and financial sector of the economy. Thus, the distinction between the real and financial factors for capital flows has been the dominant intellectual framework for classifying drivers [32]: 516–517).

There is a wide literature on the real economic determinants of FDI inflows to an emerging economy. In this context, although many economic variables have been discussed, market volume, growth rate, human capital, openness, and foreign trade balance have been the most used variables in the literature. Adhikary [1] and Kumari & Sharma [34] found market size, human capital, interest rate, and trade openness to be the key determinants of FDI inflows for Asian developing countries. Na & Lightfoot [39] indicated that labour quality and infrastructure level are important determinants of FDI inflows to China besides market size and trade openness. Gurshev [25] shows that tax rate and market size favourably influence FDI inflows to Russia. Castro et al. [10] found to be trade liberalization and domestic market dimensions as the major factors attracting FDI inflows to the Brazilian economy. Maryam & Mittal [35] determined that the market size, infrastructure facilities, labour cost, gross capital formation, exchange rate, and trade openness are basic factors for FDI inflows to Brazil, Russia, India, China, and South Africa (BRICS) countries. Cieslik [14] argues that human capital endowment, physical capital endowment, and market size encourage FDI inflow to Poland. In the context of emerging countries, Cieslik and Tran [15] are found geographical distance, skilled labour abundance, trade cost, investment cost, and market size to be the key determinants of FDI movements. Galego [23] examined the factors affecting foreign direct investment flows to the Central and Eastern European Countries. Empirical results indicate that international investments are mainly determined by such characteristics as potential demand, openness to world trade, and lower relative labour compensation levels. Janicki [27] focussed on the determinants of FDI in nine emerging European countries, specifically Bulgaria, Czech Republic, Estonia, Hungary, Poland, Slovakia, Slovenia, Romania, and Ukraine. The most important determinant of FDI was found to be trade openness, market size, and labour cost. Ghazi [24] aims to figure out the determinant of foreign direct investment in Indonesia from the year 2011 to 2016. The result of this research is that the variable degree of openness is the only variable that is significant in affecting foreign direct investment, which has a negative value.

Apart from real economic factors, many studies in the literature determine many financial factors influencing FDI flows into an economy. According to these studies, financial markets can play a vital role as much as a real economic system in attracting FDI to the host country. Many studies show that a well-developed financial system acts as a catalyst to increase FDI inflow into developing economies. The financial system of a country includes the banking sector and the stock market. An efficient banking sector can contribute to FDI inflows by providing lower costs loans availability. The efficient stock market provides foreign investors better gains from "public offering." In this regard, it is significant to identify financial system variables that contribute to FDI inflows.

Naceur et al. [40] especially determined that the existence of strong equity market is important to attract foreign capital inflows enough. On the other hand, foreign enterprise needs a developed banking system since an inefficient banking system will have high costs of operations which will be passed on to its clients resulting in high costs of capital for clients. Agbloyor et al. [3] explored the causality links between the banking system and foreign direct investment in 42 countries. Their results suggest that a more advanced banking system can lead to more FDI flows. Kholdy and Sohrabian [30] examined the causal link between FDI and financial development in developing countries. They suggest that the development of financial institutions in a country can attract more FDI. Dutta and Roy [17] study the relationship between FDI and financial development by using a panel of 97 countries. They found that developed financial markets are necessary to capture and utilize the benefits of FDI. Hajilee and Nasser [26] investigate the impact of financial market development on FDI for 14 Latin American countries over the period of 1980–2010. Their empirical findings provide supporting evidence that a well-developed financial sector attracts more FDI. Therefore, a better functioning financial market is critical for determining the amount of FDI inflows to Latin American countries. Choong et al. [12] study the relationship among FDI, financial markets, and economic growth in Malaysia from 1970 to 2001. They argue that economies with better-developed financial markets are able to benefit more from FDI to promote their economic growth. Kaur et al. [29] indicated that a developed financial system is one of the important determinants of FDI inflows to BRIC countries. They analysed the impact of financial system development on FDI with respect to BRIC countries for the period 1991 to 2010. Empirical results conclude that FDI inflows to BRIC countries are influenced by the banking sector and stock market variables. Agarwal and Mohtadi [2] examined the role of the financial market in attracting FDI in 21 developing countries over the period 1980–1997. Their analysis reveals that FDI as a ratio of gross domestic product (GDP) is positively correlated with financial indicators, including both stock market and banking variables. Nasser and Gomez [42] analysed determinants of FDI flows to 15 Latin American countries. Empirical results show that financial factors have significant role in attracting FDI. Thus, they concluded that the financial system indicators related banking sector and capital market have a strong influence on FDI inflows to Latin American countries. Nkoa [9] investigated the impact of financial factors on foreign direct investment in 52 African countries from 1995 to 2015. The econometric results support the positive and significant influence of certain financial variables on FDI flows to Africa.

In the framework of financial factors affecting global FDI movements, some studies in the literature also add the effect of monetary policy on financial indicators to analyses. Thus, this part of the literature has also indicated the interaction between monetary policy and financial indicators affecting FDI inflows to host countries. According to these studies, changes in financial indicators depending on monetary policies play a big role not only in domestic investments but also in the realization of foreign direct investments. In other words, monetary policy preferences and their effects on financial indicators can be one important determinant in the investment decisions of domestic entrepreneurs, and they can undoubtedly significantly affect the investments made by multinational companies.

When the related literature is examined, it is seen that monetary policy practices affect foreign direct investments, especially with the changes they create on financial variables such as interest rate and stock market index. Thus, it can be argued that interest rate and stock market index determined by the monetary policy are two important financial indicators that all entrepreneurs consider when making investment decisions. In this context, expansionary monetary policies implemented after the 2008 economic crisis and during the pandemic had increased the liquidity in global markets and therefore created significant positive effects on foreign direct investments by decreasing interest rates and increasing the stock market index.

Based on the findings indicated above, it is possible to examine the literature examining the effects of expansionary monetary policies applied before and during the COVID-19 on foreign direct capital inflows to developing countries under two groups. In the framework of classical investment theory, the first group examining the interest rate channel implies that the expansionary monetary policy of the central bank reduces the cost of capital lowering the interest rate, and thus promoting global foreign direct investment. The second group analysing the asset price channel uses Tobin's q theory. The expansionary monetary policy of the central bank increases the asset prices promoting the demand for assets in the stock market, and thus promoting global foreign direct investment. In summary, the recent literature focussing on the impact of expansionary monetary policies on FDI inflows to emerging markets considers two main channels: the interest rate channel and the asset price channel.

Considering the interest rate channel, classical economic approach asserts that one of the most important financial variables affecting the advantage of investing overseas is the cost of capital or interest rate for MNCs. In general, the results of economic research highlight the profit expectations of entrepreneurs as the most important factor affecting the investment level. In cases where the economic environment offers high-profit expectations for entrepreneurs, it is seen that the investment level in the country increases. Thus, it is not possible to increase private sector investments in an environment where high-profit expectations for entrepreneurs are not met. Economists from the classical economics tradition also argue that while creating high-profit expectations, entrepreneurs focus on low investment costs. In other words, the classical approach assumes that entrepreneurs try to maximize profits by minimizing costs. Thus, low-interest rates in the economy can reduce the financing cost of the investment and therefore the total costs, raising the profit expectations of the entrepreneurs and directing them to invest more. When this fact is evaluated together with monetary policy by which the central banks control money supply and credit in order to adjust the interest rate, it is clearly pointed out that the central bank’s expansionary policy significantly influences multinational companies' investment decisions.

Fornah and Yuehua [22] aim to develop an empirical framework to determine the effect of interest rate on FDI inflows to Sierra Leone using time series data for the period 1990–2016. The results showed that interest rates have a significant impact on FDI inflows and thus concluded that interest rates can be used for policy-making purposes in FDI inflows. Okafor [44] focussed on the impact of pull factors on capital movement in Nigeria using the ordinary least square (OLS) estimation technique. The result shows that interest rate and real exchange rate are key determinants of foreign direct investment in Nigeria. The result suggests that policymakers should strive to improve the financial condition by lowering the interest rate to encourage the flow and benefits of foreign direct investment in Nigeria. Fazira and Cahyadin [20] analysed the impact of interest rates on FDI inflows to Indonesia, Singapore, Malaysia, Thailand, Philippines, and Vietnam between 2004 and 2016. This research concluded that interest rate had a positive and significant impact on FDI. The recommendation of this research was the governments of Asian countries manage interest rates to attract FDI. Faroh and Shen [19] aimed to examine the impact of interest rate on FDI flow in Sierra Leone, using econometrics techniques to run multiple regression time-series data for the period of 1985 to 2012. Interest rates were found to be insignificant factors causing the variability of FDI flows. Thus, it is concluded that a high-interest rate has no effect on FDI flow in Sierra Leone. Shahzad and Zahid (2011) conducted to investigate the determinants of FDI inflow into Pakistan from 1991 to 2010. The empirical results show that interest rate is important and helpful for the decision-making of the investment policy. Accordingly, to increase more FDI into Pakistan, authorities of the country need to ensure a stable and low-interest rate level. By using annual time series data for the period 1970–2016, Musyoka and Ocharo [38] employed the OLS technique to determine the effect of real interest rate and exchange rate on FDI in Kenya. Empirical results show that real interest rates and exchange rates have a negative and significant influence on FDI inflows into Kenya. Thus, the study concluded that interest rate policy has a significant influence on attracting foreign direct investment inflows into Kenya. Singhania and Gupta [51] tried to find the best-fit model to explain variation in FDI inflows into India during the years 1995‐1997. The authors tested for various assumptions taken before applying autoregressive integrated moving average (ARIMA) using standard tests and quantified FDI policy changes using dummy variables. It was found that interest rate has an insignificant impact on FDI inflows into India.

Regarding the asset price channel, economists argue that the positive effect of monetary expansion on direct physical investments can also arise from the stock market index. Due to the increasing demand for stock markets with monetary expansion, the stock market index increases and makes it more profitable for both domestic and foreign entrepreneurs to turn to real investments. Tobin theorized this fact in the framework of the “q ratio”. The q ratio can be defined as the “Market Value of Firm's Capital Stock” divided by the “Replacement Cost of the Capital Stock”. The increase in the market value of firms according to the replacement costs of their capital causes the q ratio to be higher than 1. This indicates that the installed capital value exceeds the replacement cost in the market, resulting in higher returns. Thus, the more value the q ratio gets above 1, the more firms are willing to add to their installed capital stock. Undoubtedly, the value of q ratio greater than one depends on the increase in the stock value of the investment in the stock market. That means the firm makes more investments if the value of its stock market increases. When this phenomenon is evaluated together with the policies implemented before and during the pandemic, it can be concluded that the expansionary monetary policies implemented before and during the pandemic period had a positive effect on FDI inflows to emerging markets by increasing the stock market index.

Batten and Vo [6] found that capital markets can play an important role in determining the movement of cross-border mergers and acquisitions (M&A), which constitute an important part of FDI. Thus, the study confirms the existence of linkages between stock markets and FDI. Tsaurai [54] investigated the causality link between FDI and stock market development in Zimbabwe. Using data spanning from 1988 to 2012 based on the bi-variate causality test framework, this study discovered that there exists a long-run relationship between stock market development and FDI net inflows in Zimbabwe. Kariuki [28] examined the factors that influence foreign direct investment (FDI) flows into African countries. Annual data from 1984 until 2010 using 35 African countries are used for this panel study. Estimation results show that there is a positive and significant relationship between the stock price index performance and FDI inflows. Thus, it concluded that the good performance of stock markets has a positive and significant impact on FDI inflows. Chousa, Tamazian, and Vadlamannati [13] examined the impact of the capital market on FDI inflows to nine emerging economies from 1987 to 2006. They found a strong positive relationship between the development and quality of capital markets and M&A flows in emerging economies. Empirical evidence showed that greater efficiency of domestic capital markets encourages foreign investors and attracts international M&A. Feridun, Sawhney and Jalil [21] explored the existence of a long-term relationship between stock prices and business investment decisions in Turkey for the period 1987:01-2006:03. They prove the existence of a one-way positive causal relationship from stock prices to real business investments. Arcabic et al. [5] aim to investigate the existence and characteristics of both the long- and short-term relationships between FDI and the stock market in Croatia. The long-term results suggest the lack of connection between FDI and economic growth in Croatia. They show that in the short run, upward movement on the stock market positively affects Croatian FDI stock. That means the stock market did prove to be an important short-term determinant of FDI in Croatia. Ahmed and Malik [4] analysed the determinants of FDI inflow in Pakistan by using monthly time series data from 2003 to 2011. Results suggest that the capital market has a significant role in attracting FDI for the Pakistan economy. Thus, in order to get desirable level of FDI inflow to Pakistan, the government should ensure the operation of an efficient capital market.

## Data and methodology

In this section, we empirically examine the effects of expansionary monetary policies implemented before and during the COVID-19 on FDI inflows to developing countries. The effects of expansionary monetary policies on FDI inflows to developing countries will be determined through changes in financial indicators such as interest rates and stock market indices. Accordingly, we will try to empirically reveal the effects of financial variables on FDI inflows to emerging markets by using international data for four developing countries such as Brazil, China, Turkey and Poland for the period 2008–2021.

In the study, the data of the four countries that attract the most foreign direct investment in their regions, representing the developing countries in different continents of the world, were used. In this context, data from China, Brazil, Poland and Turkey, which attract the most foreign direct investment in their regions, were included in the analysis, representing the regions of Asia, Latin America, Europe and the Middle East, respectively [55]. The research period covers the process of expansionary monetary policies that started with the global economic crisis in 2008 and accelerated with the COVID-19 period. Due to the abundance of liquidity created in this period when expansionary monetary policies were implemented, there were great decreases in interest rates at the global level. On the other hand, the demand for a wide variety of assets increased due to the abundant liquidity. In this context, the demand for stocks in the global stock markets has increased, and as a result, the stock market indexes have increased. Thus, because of the expansionary monetary policies implemented before and during the pandemic, very positive financial conditions have been created for DFI to developing countries.

The financial indicators such as interest rate and stock market index in determining the effects of expansionary monetary policies on FDI have very strong theoretical justifications within the framework of classical investment theory and Tobin's Q theory, respectively. The classical investment theory argues that entrepreneurs give more importance to low investment costs while creating high-profit expectations. Thus, the low-interest rates in the economy reduce the financing cost of the investment and therefore the total costs, which raises the profit expectations of the entrepreneurs and directs them to invest more. Tobin's Q theory asserts that the Q ratio, which equals the stock market value of a company divided by its assets' replacement cost, is significant in taking new investment decisions for companies. High stock prices causing higher Tobin's q values encourage companies to invest more in physical capital. Because, in this case, physical capital investments gain "worth" in the stock markets more than the price companies paid for them.

In our study, the effect of financial indicators on FDI was basically estimated within the framework of Model-1 and as shown in Eq. 1.

1 $$\ln fdi_{t} = \beta_{0} + \beta_{1} \ln {\text{stock}}p_{t} + \beta_{2} \ln i_{t} + \beta_{3} D_{1} + \beta_{4} D_{2} + u_{t}$$

The logarithmic form of all variables was used in the analysis. The dependent variable in the model is the foreign direct investment $\left( {\ln fdi_{t} } \right)$, while the independent variables consist of global stock prices $\left( {\ln {\text{stock}}p_{t} } \right)$ and global interest rates $\left( {\ln i_{t} } \right)$. $D_{1}$ and $D_{2}$ indicate the dummy variables for the 2008 financial crisis and COVID-19 pandemic, respectively, while $u_{t}$ is the error term.

It is theoretically expected that there will be a negative relationship between FDI and interest rate and a positive relationship between the stock market index and FDI within the framework of the classical investment approach and Tobin's Q theory, respectively. Accordingly, within the framework of Model 1, it was investigated whether the implementation of expansionary monetary policy causing to decrease in interest rates and increase in stock market indexes positively affected the FDI flow towards developing countries.

We also estimated the Model-2 shown in Eq. 2 for “robustness check”. In this context, by adding a new regressor to the core regression, the effect of interest rate and stock price on FDI was re-estimated.

2 $$\ln fdi_{t} = \beta_{0} + \beta_{1} \ln {\text{stockmarkets}}_{t} + \beta_{2} \ln i_{t} + \beta_{3} \ln gdp_{t} + \beta_{4} D_{1} + \beta_{5} D_{2} + u_{t}$$

In Model-2, the regression specification in Model-1 has been modified by adding a new real economic indicator such as Gross Domestic Product (lngdp). Thus, we also tested how precisely the coefficients of financial variables estimate the impact of expansionary monetary policies on FDI inflows to developing countries. Theoretically, there is a positive relationship between national income and investments. The Keynesian approach argues that when calculating the profit expectations of entrepreneurs, it is more important to maximize expected revenue rather than cost minimization. Accordingly, in a situation where the national income increases, the demand in the market is revived due to consumer income. Such a situation increases the revenue and profit expectations of entrepreneurs and leads them to make more physical investments.

In the estimation of Model-1 and Model-2, the quarterly frequency data set between 2008:Q4-2021:Q3 covering the post-2008 economic crisis and the pandemic period was used. The FDI variable is used as the ratio of inward foreign direct investment to the country’s GDP. Data on the FDI variable of all countries were obtained from The Organisation for Economic Co-operation and Development (OECD) database. The Standard & Poor's (S&P) 500 index is preferred as the indicator of the global stock markets variable. The S&P 500 was introduced in 1957 by Standard & Poor's, as a stock market index to track the value of 500 large corporations listed on the New York Stock Exchange. The US 10-year government bond interest rate is chosen as the indicator of global interest rates the second independent variable. This ratio also indicates the unconventional monetary policy implemented by FED after the 2008 financial crisis. The datasets of S&P 500 and US 10-year government bond interest rates were obtained from St. Louis FED [52, 53]. GDP variables (current, $) are also sourced from The Organisation for Economic Co-operation and Development (OECD) database.

The sample of the study includes emerging markets including Brazil, China, Turkey, and Poland. The countries examined were chosen to represent developing countries on different continents of the world based on the availability of data.

Table 1 shows the descriptive statistics of the data. All variables have low standard error. Although the lnfdi series of countries are almost similar, the most fluctuation is in Poland.

**Table 1:** Descriptive statistics

	Obs	Mean	Maximum	Minimum	Std. Dev
lnstockp	52	7.55	8.39	6.59	0.43
lni	52	0.77	1.31	− 0.43	0.43
lnfdi (Brazil)	52	1.18	1.75	0.51	0.34
lnfdi (China)	52	0.75	1.53	− 0.33	0.47
lnfdi (Poland)	52	1.04	2.55	− 3.86	0.91
lnfdi (Turkey)	52	0.35	1.12	− 0.98	0.37
lngdp (Brazil)	52	15.18	16.25	14.25	0.63
lngdp (China)	52	18.49	19.09	17.73	0.37
lngdp (Poland)	52	14.25	14.75	13.69	0.29
lngdp (Turkey)	52	14.33	16.56	12.84	1.12

In our study, the effects of independent variables on foreign direct investments for developing countries are estimated by ARDL methodology. Pesaran and Pesaran [45], Pesaran and Shin [46], and Pesaran et al. [47] developed the ARDL framework. ARDL method was preferred because it offers two important advantages over other time-series analyses. Firstly, this approach, unlike traditional cointegration tests, provides the opportunity to estimate even if the series has different degrees of integration. Secondly, reliable and consistent results can be acquired even in the estimations obtained with small samples.

There are three important stages in the ARDL methodology. Initially, the stationarity of the time series is checked. Because the series to be used in the ARDL methodology should be stationary in the level or first difference. In the second stage, the long-term interaction among the variables is examined with the cointegration test. The cointegration relationship is investigated with the Bound Test. In the last step, if there is a cointegration relationship between the variables, the coefficients showing the correlation between the variables are estimated in the long and short run.

Since the series is to be stationarity for times series analysis, it should be investigated the stationary degree of series before estimations. Within the scope of empirical analysis, first, Augmented Dickey–Fuller (ADF), Phillips–Perron (PP) unit root tests developed by Dickey and Fuller [16], Phillips, and Perron [48], respectively, examined the stationarity of the series.

Bound Test is estimated based on the Unrestricted Error Correction Model (UECM) shown in Eq. (3). In the model, ∆ represents the first difference of the series, while m indicates the optimum lag of variables. In our study, UECM is estimated with the optimum lag length to specify according to Akaike information criteria (AIC). The F or Wald test statistic is compared with the critical values calculated by Narayan [41], and it is decided whether there is a cointegration relationship.

3 $$\Delta Y_{t} = \alpha_{0} + \mathop \sum \limits_{i = 1}^{m} \alpha_{1} \Delta Y_{t - i} + \mathop \sum \limits_{i = 0}^{m} \alpha_{2} \Delta X_{t - i} + \alpha_{3} \ln Y_{t - 1} + \alpha_{4} X_{t - 1} + \varepsilon_{t}$$

After indication of the cointegration relationships among the variables, the long-term coefficients are calculated. The long-term relationship between the variables is determined by estimating the ARDL model shown in Eq. (4). In this equation, p and q represent the lag length values in the model.

4 $$Y_{t} = \beta_{0} + \mathop \sum \limits_{i = 1}^{p} \beta_{1i} Y_{t - i} + \mathop \sum \limits_{i = 0}^{q} \beta_{2i} X_{t - i } + e_{t}$$

In the last section, short-term relationships are estimated with the error correction model in Eq. (5). In this equation, $ECT_{{\left( {t - 1} \right)}}$ indicates the coefficient of error correction term. It specifies whether the impact of a shock that will cause a deviation from the long-term equilibrium tends towards equilibrium. Therefore, it is quite important for the working of the error correction mechanism.

5 $$\Delta Y_{t} = \beta_{0} + \mathop \sum \limits_{i = 1}^{p} \beta_{1i} \Delta Y_{t - i} + \mathop \sum \limits_{i = 0}^{q} \beta_{2i} \Delta X_{t - i } + \beta_{3} ECT_{{\left( {t - 1} \right)}} + e_{t}$$

## Empirical results

The stationarity of the series is investigated with ADF and PP unit root tests, and the results are presented in Table 2. Two separate model estimations including constant and constant + trend are used to perform ADF and PP unit root tests. Both ADF and PP unit root tests have shown that there is no stationarity problem in the variables in terms of estimations to be made with the ARDL approach since there is no stationary series in the second difference. Accordingly, lnstockp, lni, lnfdi (China, Turkey), lngdp (Brazil, China, Poland, Turkey) are stationary at first difference. In addition, lnfdi (Brazil, Poland) are stationary at level.

**Table 2:** ADF and PP unit root tests

	ADF		PP	
	c	c + t	c	c + t
lnstockp	− 1.118 (1)	− 3.567** (0)	0.241 (18)	− 3.522** (2)
Δlnstockp	− 10.151*** (0)	− 10.057*** (0)	− 10.222*** (4)	− 10.132*** (4)
lni	− 2.251 (1)	− 3.425* (5)	− 1.927 (2)	− 2.564 (2)
Δlni	− 5.534*** (1)	− 5.474*** (1)	− 4.616*** (10)	− 4.590*** (11)
lnfdi (Brazil)	− 6.471*** (0)	− 6.533*** (0)	− 6.561*** (3)	− 6.620*** (3)
lngdp (Brazil)	0.135 (1)	− 3.551** (1)	0.549 (1)	− 2.882 (2)
Δlngdp (Brazil)	− 4.672*** (0)	− 4.699*** (0)	− 4.619*** (3)	− 4.662*** (3)
lnfdi (China)	− 1.327 (4)	− 2.837 (4)	− 3.099** (5)	− 4.388*** (4)
Δlnfdi (China)	− 7.726*** (2)	− 7.641*** (2)	− 13.445*** (18)	− 13.228*** (18)
lngdp (China)	− 1.049 (8)	− 1.975 (8)	− 0.794 (12)	− 7.601*** (9)
Δlngdp (China)	− 22.568*** (2)	− 24.360*** (2)	− 25.750*** (11)	− 29.584*** (11)
lnfdi (Poland)	− 3.878*** (2)	− 4.061 (2)	− 8.530*** (1)	− 8.780*** (0)
lngdp (Poland)	− 0.322 (9)	− 4.182*** (6)	− 0.947 (8)	− 3.848** (1)
Δlngdp (Poland)	− 4.648*** (8)	− 4.578*** (8)	− 8.313*** (5)	− 8.193*** (5)
lnfdi (Turkey)	− 3.268** (5)	− 3.310* (5)	− 4.490*** (4)	− 4.449*** (4)
Δlnfdi (Turkey)	− 8.866*** (1)	− 8.846*** (1)	− 13.622*** (2)	− 13.634*** (2)
lngdp (Turkey)	3.768 (10)	− 0.106 (10)	2.788 (2)	− 1.688 (1)
Δlngdp (Turkey)	− 4.119*** (3)	− 4.878*** (4)	− 6.920*** (3)	− 7.946*** (3)

*t* statistics values are given in the table and *, **, *** indicate statistically significant at 10%, 5% and 1%, respectively. (..) shows the optimum lag lengths automatically selected according to the Akaike information criteria in the ADF test, and the Newey–West estimator in the PP test

The cointegration relationship between the variables has been investigated by using the bound test. In the framework of the bound test, Model 1 and Model 2 indicated in Eq. (1) and Eq. (2), respectively, are estimated by using the UECM model shown in Eq. (3). Empirical results of the Bound Test related to Model 1 and Model 2 are presented in Table 3. Findings show that there are cointegration relationships in both Models for four countries since the statistical values are greater than the upper table value in all cases.

**Table 3:** Bound tests

**Test statistic**	**Value**	Signif	I(0)	I(1)
F Statistic (Brazil-Model 1)	6.72	10% 5% 1%	2.2 2.56 3.29	3.09 3.49 4.37
F Statistic (China-Model 1)	5.25			
F Statistic (Poland-Model 1)	6.22			
F Statistic (Turkey-Model 1)	7.97			
F Statistic (Brazil-Model 2)	7.42	10% 5% 1%	2.08 2.39 3.06	3.00 3.38 4.15
F Statistic (China-Model 2)	4.75			
F Statistic (Poland-Model 2)	5.42			
F Statistic (Turkey-Model 2)	8.59			

After the cointegration relationship is found, we apply the ARDL technique to estimation the long-term coefficients. In this framework, Model 1 and Model 2 indicated in Eq. (1) and Eq. (2), respectively, are estimated by using the ARDL model shown in Eq. (4). Table 4 shows the results of ARDL long-term coefficients and diagnostic tests for Brazil, China, Poland, and Turkey.

**Table 4:** ARDL long-term coefficients

	Brazil		China		Poland		Turkey	
	Model 1	Model 2	Model 1	Model 2	Model 1	Model 2	Model 1	Model 2
*lnstockp*	5.17*** (1.39)	6.11*** (1.54)	2.56*** (0.51)	2.30*** (0.77)	− 0.57 (0.57)	− 1.54 (1.95)	0.47** (0.18)	1.71** (0.73)
*lni*	− 0.24 (0.52)	− 0.68 (0.59)	− 3.39*** (0.98)	− 2.69*** (0.638)	− 1.82** (0.82)	− 2.29** (1.20)	− 0. 60** (0.26)	− 0.46** (0.26)
*lngdp*		3.61*** (0.94)		− 3.18 (1.98)		1.66 (2.60)		− 0.42 (0.26)
*D*_*1*_	3.33*** (0.68)	4.52*** (1.03)	− 0.59 (1.36)	− 0.70 (1.55)	0.13 (0.75)	2.78 (1.56)	2.14*** (0.59)	2.32*** (0.53)
*D*_*2*_	0.47** (0.52)	0.43 (0.56)	− 3.49** (0.87)	− 3.49*** (0.77)	− 1.64** (0.85)	− 3.41** (1.24)	− 0.68** (0.33)	− 0.65* (0.33)
*C*	− 0.13*** (0.03)	6.07 (4.30)	− 16.65 (4.31)	19.70*** (23.86)	4.10 (4.53)	− 12.51 (23.87)	− 4.57** (1.40)	− 8.04*** (2.04)
$R^{2}$	0.63	0.57	0.87	0.93	0.36	0.66	0.62	0.71
$\overline{R}^{2}$	0.37	0.37	0.81	0.86	0.24	0.37	0.47	0.53
${\upchi }_{{\text{JB }}}^{2}$	1.79 [0.40]	1.07 [0.58]	0.39 [0.82]	0.26 [0.87]	0.03 [0.98]	0.40 [0.81]	0.08 [0.95]	0.68 [0.70]
${\upchi }_{{{\text{BPG}}}}^{2}$	0.85 [0.64]	0.67 [0.79]	0.93 [0.54]	0.60 [0.89]	0.56 [0.80]	0.46 [0.97]	0.77 [0.69]	1.19 [0.47]
${\upchi }_{{{\text{BG}}}}^{2}$	0.95 [0.44]	0.59 [0.55]	0.08 [0.98]	0.72 [0.49]	1.10 [0.36]	0.16 [0.85]	0.30 [0.87]	0.76 [0.47]
${\upchi }_{{{\text{RAMSEY}}}}^{2}$	0.29 [0.59]	0.98 [0.32]	0.34 [0.56]	0.04 [0.83]	1.62 [0.20]	1.94 [0.16]	0.01 [0.89]	0.03 [0.85]

*, **, *** indicate statistical significant at 10%, 5% and 1%, respectively. (..) shows the standard errors and [..] presents probability value

The estimation results obtained within the framework of Model 1 were generally compatible with theoretical expectations. Accordingly, it is observed that interest rates (lni) in the before and during COVID-19 when expansionary monetary policies were implemented, within the framework of classical investment theory, increased foreign direct capital inflows (lnfdi) in China, Poland and Turkey, except Brazil. The effect of interest rates on capital inflows in Brazil was not statistically significant. Interest rates made the most significant contribution to foreign direct capital inflows in China during the period when expansionary monetary policies were implemented. Poland was the second country benefiting from abundant money and low-interest rates. Turkey, on the other hand, was the country that benefited the least from the interest rate advantage in terms of foreign direct investment. On the other hand, within the framework of Tobin's Q theory, it is determined that the rising stock market index during the implementation of expansionary monetary policies positively affected foreign direct capital inflows in China, Brazil and Turkey, except Poland. In Poland, the effect of stock market index (lnstockp) on foreign capital inflows (lnfdi) was not statistically significant. The stock market index made the most significant contribution to foreign direct capital inflows in Brazil during the implementation of expansionary monetary policies. The second country benefiting from the stock market index was China. Turkey, on the other hand, was the country that benefited the least from the interest rate advantage in terms of foreign direct investment.

The results in Model 2 estimated for robustness check were similar to the original Model 1. Thus, the robustness of the results obtained from the original Model 1 was demonstrated. The results in Model 2 showed that, similar to Model 1, interest rates (lni) increased foreign direct capital inflows (lnfdi) in China, Poland and Turkey, except for Brazil. Again, while the interest rates had the greatest impact on China, the least impact was on Turkey. In addition, in the estimation of Model 2, the rising stock market index during the implementation of expansionary monetary policies positively affected foreign direct capital inflows in China, Brazil and Turkey, excluding Poland. The GDP control variable included in Model 2 for robustness testing was insignificant in all countries except Brazil.

**Fig. 1:**
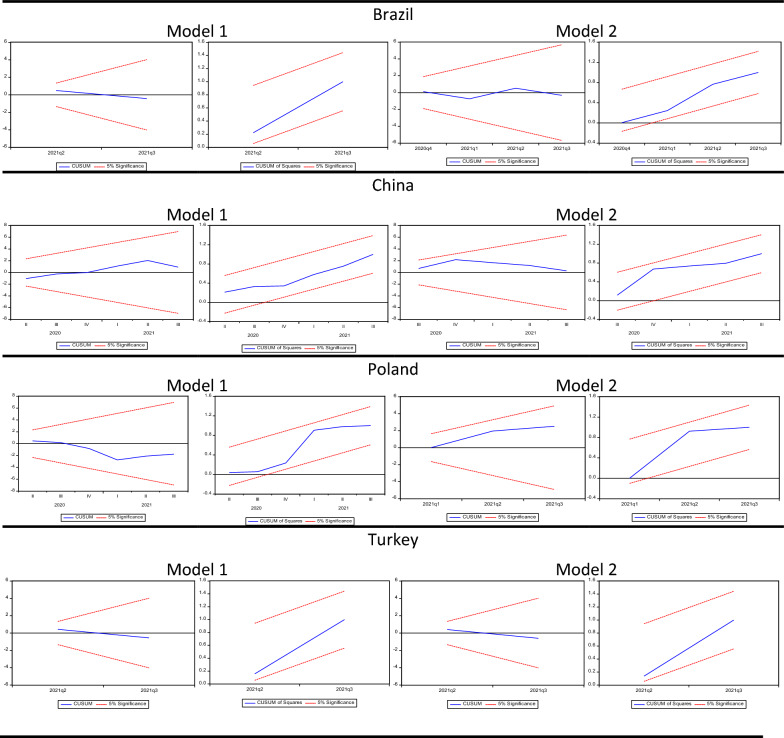
CUSUM and CUSUMSQ graphs

Concerning the diagnostic tests related to the estimations, the results of the Jarque–Bera normality test $\left( {\chi_{{{\text{JB}}}}^{2} } \right)$ show that the error terms are normally distributed since the null hypothesis is accepted for each estimation. It is performed Breusch–Godfrey LM test $\left( {\chi_{{{\text{BG}}}}^{2} } \right)$ for autocorrelation and according to the test result, the null hypothesis, that is no serial correlation, is accepted. In the Breusch–Pagan–Godfrey heteroscedasticity test $\left( {\chi_{{{\text{BPG}}}}^{2} } \right)$, the null hypothesis that the variance of error terms is constant, is tested. As a result, there is no problem of heteroscedasticity in the estimations. It is also examined whether there is a specification error in the functional form of the model with the Ramsey-Reset test. The null hypothesis that there is no specification error is accepted. Finally, Fig. 1 presents the CUSUM and CUSUMSQ graphics for all models. CUSUM and CUSUMQ tests show that the parameters are stable and there are no structural changes in all models.

In general, Model 1 and Model 2 short-term forecast results were in harmony with long-term forecasts in terms of the stock market index, but it was not possible to reach the same conclusion in terms of interest rates. The short-term estimation results are in Table 5. Thus, it can be concluded that the effect of the stock market index on capital inflows is strong in both the short and long run. However, it can be said that the relationship between the interest rate and capital inflows is more valid in the long-run than in the short run.

**Table 5:** Estimation results of error correction model

Variables	Brazil		China		Poland		Turkey	
	Model 1 (4,0,4,4,4)	Model 2 (3,2,0,0,4,2)	Model 1 (4,4,3,2,0)	Model 2 (2,4,3,4,4,4)	Model 1 (3,0,1,0,0)	Model 2 (3,3,4,2,4,3)	Model 1 (2,4,0,4,1)	Model 2 (2,4,3,4,4,4)
D (lnfdi(-1))	1.00*** (0.26)	0.35** (0.14)	− 0.23** (0.10)	− 0.19 (0.11)	0.14 (0.17)	0.25 (0.18)	0.43*** (0.12)	0.44*** (0.11)
D (lnfdi(-2))	0.81*** (0.19)	0.18 (0.11)	− 0.26** (0.10)		0.30** (0.11)	0.39*** (0.12)		
D (lnfdi(-3))	0.45*** (0.13)		− 0.37*** (0.09)					
D (lnstockp)		8.60*** (2.05)	− 4.02*** (1.15)	− 2.58** (1.04)		5.47* (3.12)	6.39*** (0.95)	6.95*** (0.96)
D (lnstockp(-1))		− 6.87*** (2.04)	− 5.17*** (1.19)	− 7.72*** (1.40)		14.02*** (3.28)	1.74* (0.88)	1.92** (0.86)
D (lnstockp-2))			− 5.82*** (1.13)	− 5.89*** (1.30)		10.18*** (3.23)	3.14*** (0.80)	2.89*** (0.81)
D (lnstockp-3))			− 5.45*** (1.06)	− 4.27*** (1.07)			2.30*** (0.71)	2.97*** (0.73)
D (lni)	0.38 (1.21)		− 2.26*** (0.48)	− 1.72*** (0.52)	0.31 (1.14)	2.50 (1.65)		
D (lni(-1))	− 1.27 (1.23)		1.76*** (0.57)	1.27** (0.57)		1.60 (1.85)		
D (lni(-2))	1.47 (1.21)		1.03* (0.53)	1.60*** (0.56)		0.90 (1.55)		
D (lni(-3))	2.23** (1.03)					3.76 (1.99)		
D (lngdp)				7.54*** (1.23)		3.36 (3.26)		1.92*** (0.56)
D (lngdp(-1))				6.44*** (1.46)		11.75*** (3.19)		
D (lngdp(-2))				4.53*** (1.23)				
D (lngdp(-3))				8.15*** (1.08)				
D (D1)	5.69*** (1.14)	4.45*** (0.95)	− 1.67*** (0.56)	− 2.00*** (0.50)		6.17*** (1.59)	1.72*** (0.42)	1.91*** (0.40)
D (D1(-1))	− 1.69*** (0.72)	− 3.29*** (0.87)	− 1.51*** (0.55)	− 1.11** (0.45)		2.01 (1.28)	− 0.15 (0.32)	− 0.03 (0.31)
D (D1(-2))	− 3.73*** (0.86)	− 4.04*** (0.84)		0.30 (0.39)		− 0.40 (1.19)	− 1.33*** (0.36)	− 1.34*** (0.34)
D (D1(-3))	− 3.25*** (0.98)	− 2.12** (0.81)		0.89** (0.38)		− 5.12*** (1.08)	− 1.23*** (0.36)	− 1.34*** (0.34)
D (D2)	4.42*** (1.27)	0.93 (1.05)		− 0.00 (0.52)		− 0.02 (1.58)	0.89** (0.39)	1.24*** (0.38)
D (D2(-1))	− 2.45* (1.31)	− 6.17*** (1.12)		− 0.24 (0.66)		7.37*** (2.23)		1.00** (0.39)
D (D2(-2))	− 0.48 (1.35)			0.39 (0.66)		3.96* (2.03)		0.94** (0.41)
D (D2(-3))	3.20** (1.40)			2.11*** (0.60)				1.00** (0.40)
ECT (-1)	− 1.83*** (0.27)	− 1.64*** (0.21)	− 0.57*** (0.09)	− 0.73*** (0.11)	− 1.33*** (0.20)	− 1.52*** (0.22)	− 0.68*** (0.12)	− 1.24*** (0.14)

*, **, *** indicate statistically significant at 10%, 5% and 1%, respectively. (..) shows the standard errors

Model-1 and Model-2 short-term estimation results of the stock market index are statistically significant to a large extent. In addition, developments in the stock market index, in line with theoretical expectations, affect capital inflows positively, except for China. Thus, it can be said that short-term forecasts are generally compatible with long-term forecast results. Model-1 short-term estimation results indicate that the estimation results showing the relationship between the stock market index and capital inflow in China and Turkey are statistically significant. However, while the increases in the stock market index positively affect capital inflows to Turkey, it is determined that it has the opposite effect in China. The short-term estimation results of Model 1 stock market index could not be determined in Brazil and Poland. Model-2 short-term estimation results determined that the estimation results for the stock market index in all countries are statistically significant. In addition, developments in the stock market index positively affect capital inflows in all countries except China. Similar to the results of Model 1, increases in the stock market index in China affect capital inflows negatively in Model 2.

Short-term estimates of the interest rate, on the other hand, are not statistically significant to a large extent within the framework of both Model 1 and Model 2. Only in China, the interest rate values are statistically significant in the short-term forecasts made within the framework of Model 1 and Model 2. This situation revealed that the short-term and long-term forecast values for interest rates do not match. Therefore, it can be said that the relationship between the interest rate and capital inflows is more valid in the long-run than in the short run.

Finally, estimation results of ECT indicate that the speed of adjustment is computed simply as a percentage of the equilibrium error that is corrected each period. Coefficients of ECT are negative and statistically significant. The results from error correction models also show that changes in variables of models the short-term dynamic adjustment path of FDIs towards their long-run equilibrium levels. In addition, it can be said that the speed of adjustment towards the equilibrium is rapid for China and Turkey and is in the form of decreasing fluctuations for Brazil and Poland.

## Discussions

Overall, the evidence from time-series analysis revealed that interest rate and stock prices play an important role in FDI inflows to four developing countries before and during the COVID-19. In other words, the expansionary monetary policies implemented before and during the COVID-19 had significant effects on foreign direct investments with the changes they created in financial indicators such as interest rate and stock market prices. More specifically, in this period when liquidity was plentiful, falling interest rates, within the framework of classical investment theory, reduced investment financing costs and positively affected the volume of foreign direct investments. In addition, the liquidity abundance has also increased the stock market indexes, creating a very positive environment for foreign direct investment within the framework of Tobin's Q theory.

When the effects of the interest rate and stock price are compared, it has been revealed that the effect of the stock market index on capital inflows to developing economies is more important than the effect of interest rates. In other words, expansionary monetary policies have had an impact on capital inflows to developing countries more through the stock market index than the interest rate. Because the coefficients of the stock market index were found to be higher than the coefficients of the interest rates. In addition, the positive effect of stock market indices on capital inflows has been determined in both long- and short-term forecasts. However, the positive effect of the interest rate is mostly revealed in long-term forecasts. Short-term estimates of the impact of interest rates, on the other hand, were generally statistically insignificant.

The results of the study also enabled us to make some inferences about the possible effects of contractionary monetary policies implemented in the post-COVID-19 period on FDI flows to developing countries. In the last period, with the loss of importance of the COVID-19 and inflation increases, it seems that most of the central banks have started to return to contractionary monetary policies. In other words, both the weakening of the pandemic and the inflation problem in the recent period forced Central Banks to implement a contractionary monetary policy in the post-COVID-19 period. Accordingly, it is expected that interest rates will increase and stock market indexes will decrease in all countries in the post-COVID-19 period. Therefore, favourable financial conditions for foreign direct capital inflows to developing countries will probably disappear in the post-COVID-19 period.

The results obtained allow important implications for policy design towards attracting FDI. In general, policymakers in developing countries should pay special attention to attracting foreign direct investment to offset the negative impact of interest rate and stock price resulting from the post-COVID-19 contractionary monetary policy. In this context, some policies towards the real economy can be implemented in order to ensure that foreign direct investments to developing countries continue uninterruptedly in the post-COVID-19 period. In this context, as frequently mentioned in the literature, improving the physical infrastructure of energy, transportation and communication [33], increasing the level of incentives for foreign investments, including corporate tax reductions and investment subsidies [56], and enhancing the skilled workforce [36] may be important policy options. In addition, the development of legal and institutional arrangements that support the functioning of the free market [49], the development of rules and controls for the protection of intellectual property rights [31], and the strengthening of the political and social stability of the country [18] can be preferred as other policy options.

## Conclusion

The determination of the expansionary monetary policies implemented before and during COVID-19 on FDI flows to emerging economies is crucial to design policies in post-pandemics period to attract FDI in emerging markets. Accordingly, this study analyses the effect of monetary expansion before and during pandemics on foreign direct investment inflow to emerging market economies. ARDL method has been used to present evidence of the relationship between foreign direct investment inflows and financial variables for four emerging markets including Brazil, China, Turkey, and Poland during the period from 2008 to 2021.

The empirical analysis examined the effect of the expansionary monetary policy applied before and during the COVID-19 on foreign direct investments in emerging economies, through two financial channels such as the interest rate and the stock market index. In the framework of classical economic approach, the interest rate channel implies that the expansionary monetary policy of the central bank reduces the cost of capital lowering the interest rate, and thus promoting global foreign direct investment. The asset price channel works under Tobin's q theory. The expansionary monetary policy of the central bank increases the asset prices promoting the demand for assets in the stock market, and thus promoting global foreign direct investment.

The evidence from time-series analysis revealed that interest rate and stock prices play an important role in FDI inflows to developing countries before and during the COVID-19. When the effects of the interest rate and stock price are channels compared, it seems that the effect of the stock market index on capital inflows to developing economies is more important than the effect of interest rates. Thus, during the research period when expansionary monetary policies were implemented, the stock market index channel, which was put forward within the framework of Tobin's Q theory, worked more strongly on FDI inflows to emerging economies.

The empirical findings of the study are not only important for illustrating the fundamental dynamics of capital flows before and during COVID-19, but also provide important policy implications for continued FDI inflows into emerging economies in the post-COVID-19 era. So much so that today, with the decrease of the effect of COVID-19 and the increase in inflation, central banks all over the world are starting to return to contractionary monetary policies. Of course, tightening monetary policy in the post-COVID-19 era will bring notable changes in the impact of interest rate and stock price on FDI flows to developing countries. Therefore, it can be expected that favourable financial conditions for foreign direct capital inflows to developing countries will disappear in the post-COVID-19 period. In this period, it will not be possible for developing countries to attract foreign capital to their economies by taking advantage of low-interest rates and high stock prices.

In terms of policy inference, policymakers in emerging economies should focus more on policies that attract FDI in order to eliminate the disadvantages of financial indicators resulting from the contractionary policy implemented in the post-COVID-19 era. Therefore, policy-makers in developing countries should pay special attention to policies aimed at improving factors other than financial indicators that affect FDI inflows. These policies may include improving physical infrastructure, strengthening human capital, tax cuts and investment incentives to increase the profitability of investment in the country. In addition, such policies may include legal and institutional arrangements that will ensure the effective functioning of the free market, and practices aimed at ensuring political and social stability.

## Data Availability

The datasets used and/or analysed during the current study are available from the corresponding author on reasonable request.

## Abbreviations

FDI: Foreign direct investment
ARDL: Autoregressive distributed lag
FED: The Federal Reserve System
BRICS: Brazil, Russia, India, China, and South Africa
GDP: Gross domestic product
OLS: The ordinary least square
ARIMA: Autoregressive integrated moving average
M&A: Mergers and acquisitions
S&P: Standard & Poors
OECD: The organisation for economic co-operation and development
ADF: Augmented Dickey–Fuller
PP: Phillips–Perron
UECM: Unrestricted error correction model
AIC: Akaike information criteria
ECT: Error correction term
